# Acute cardiotoxicity after initiation of the novel tyrosine kinase inhibitor gilteritinib for acute myeloid leukemia

**DOI:** 10.1186/s40959-021-00122-x

**Published:** 2021-10-22

**Authors:** Lisa Kim, Brian Fowler, Courtney M. Campbell, Jeremy Slivnick, Haseeb Nawaz, Yaquta Kaka, Patrick Ruz, Ajay Vallakati, Ragavendra Baliga, Sumithira Vasu, Daniel Addison

**Affiliations:** 1 Cardio-Oncology Program, Division of Cardiology, Department of Internal Medicine, The Ohio State University Medical Center, Davis Heart and Lung Research Institute, 473 W. 12th Ave., Columbus, OH 43210 USA; 2 Bone Marrow Transplantation and Cellular Therapies Program, Division of Hematology, The Ohio State University James Comprehensive Cancer Center, Columbus, OH USA; 3 Division of Cancer Control and Prevention, James Cancer Hospital and Solove Research Institute at The Ohio State University, Columbus, OH USA

**Keywords:** Gilteritinib, FLT3-mutation, Cardio-oncology, Acute myeloid leukemia, Cardiac MRI

## Abstract

**Background:**

Gilteritinib is a novel FMS-like tyrosine kinase 3 inhibitor recently approved by the United States Food and Drug Administration in 2018 for relapsed or refractory acute myeloid leukemia. However, gilteritinib may be associated with underrecognized cardiotoxicities.

**Case presentation:**

This case describes a patient with a history significant for hyperlipidemia who was diagnosed with relapsed acute myeloid leukemia. After four doses of gilteritinib monotherapy, she abruptly developed acute systolic heart failure with global hypokinesis and septal wall motion abnormalities. Two days after discontinuation, cardiac magnetic resonance imaging showed partial recovery of her left ventricular ejection fraction as well as myocardial edema and non-ischemic fibrosis suggestive of inflammatory cardiomyopathy. She underwent intravenous diuresis and eventually started guideline-directed heart failure therapy. Follow-up cardiac magnetic resonance imaging five months later showed improved ejection fraction with mild non-ischemic fibrosis and resolution of myocardial edema and inflammation. She later received an allogeneic stem cell transplant from a matched unrelated donor.

**Conclusions:**

Gilteritinib may be associated with early cardiotoxicities, including non-ischemic cardiomyopathy and myocarditis. Cardiac magnetic resonance imaging can be an important modality to help differentiate or diagnose early cardiotoxicities associated with novel targeted therapies.

**Supplementary Information:**

The online version contains supplementary material available at 10.1186/s40959-021-00122-x.

## Introduction

Constitutive activation of FMS-like tyrosine kinase 3 (FLT3) occurs in approximately 30% of acute myeloid leukemias (AML) and is clinically associated with an aggressive disease course and higher rates of relapse [1, 2]. FLT3 inhibitors have shown promising antileukemic activity in recent clinical trials. Gilteritinib, a novel tyrosine kinase inhibitor of FLT3, was rapidly approved by the United States Food and Drug Administration (FDA) in 2018 after the multicenter, randomized phase III ADMIRAL trial which demonstrated significantly higher overall survival and response rates in comparison with salvage chemotherapy in AML. This trial established gilteritinib monotherapy as the new standard of care for relapsed or refractory FLT3-mutated AML [2]. However, gilteritinib may be associated with previously underrecognized cardiotoxicities. Here, we present a case of acute heart failure and evidence of drug-induced myocarditis in a patient with relapsed AML treated with gilteritinib, which improved upon withholding therapy. We also review other potential cardiac toxicities linked with this emerging drug class.

## Case presentation

A 56-year-old woman had a history of hyperlipidemia, osteoarthritis, gastroesophageal reflux disease, and AML in complete remission. Her home medications were meloxicam and omeprazole; she was allergic to statins and penicillins. Her AML was treated with 7 + 3 induction chemotherapy with cytarabine and daunorubicin, followed by maintenance therapy with azacitidine—stopped 10 months before this presentation. She presented to the emergency department with recurrent fevers to 102 °F, generalized myalgias, fatigue, pharyngitis, and diffuse bruising. The examination was notable for oropharyngeal exudates, cervical lymphadenopathy, and generalized ecchymosis on all extremities. She was found to have new leukocytosis to 53 K/uL with 86% circulating blasts, acute anemia to 7 g/dL, and new thrombocytopenia to 18 K/uL, concerning for AML recurrence.

Empiric vancomycin and cefepime were started for febrile neutropenia. A broad immunocompromised infectious work-up initially was unremarkable for bacterial, and fungal pathogens. Viral serologies were negative for influenza A and B, respiratory syncytial virus, parainfluenza, adenovirus, metapneumovirus, rhinovirus, enterovirus, coronavirus, coronavirus-19, cytomegalovirus, parvovirus B19, coxsackie virus A and B, and herpes simplex virus 1 and 2. Computed tomography of the chest revealed patchy nodular opacities in the mid to lower lungs with ground glass airspace disease. Eventually, a bronchoscopy was performed. The patient was diagnosed with *Serratia marcescens* pneumonia and antibiotics were deescalated to levofloxacin for a total antibiotic duration of 14 days.

**Fig. 1:**
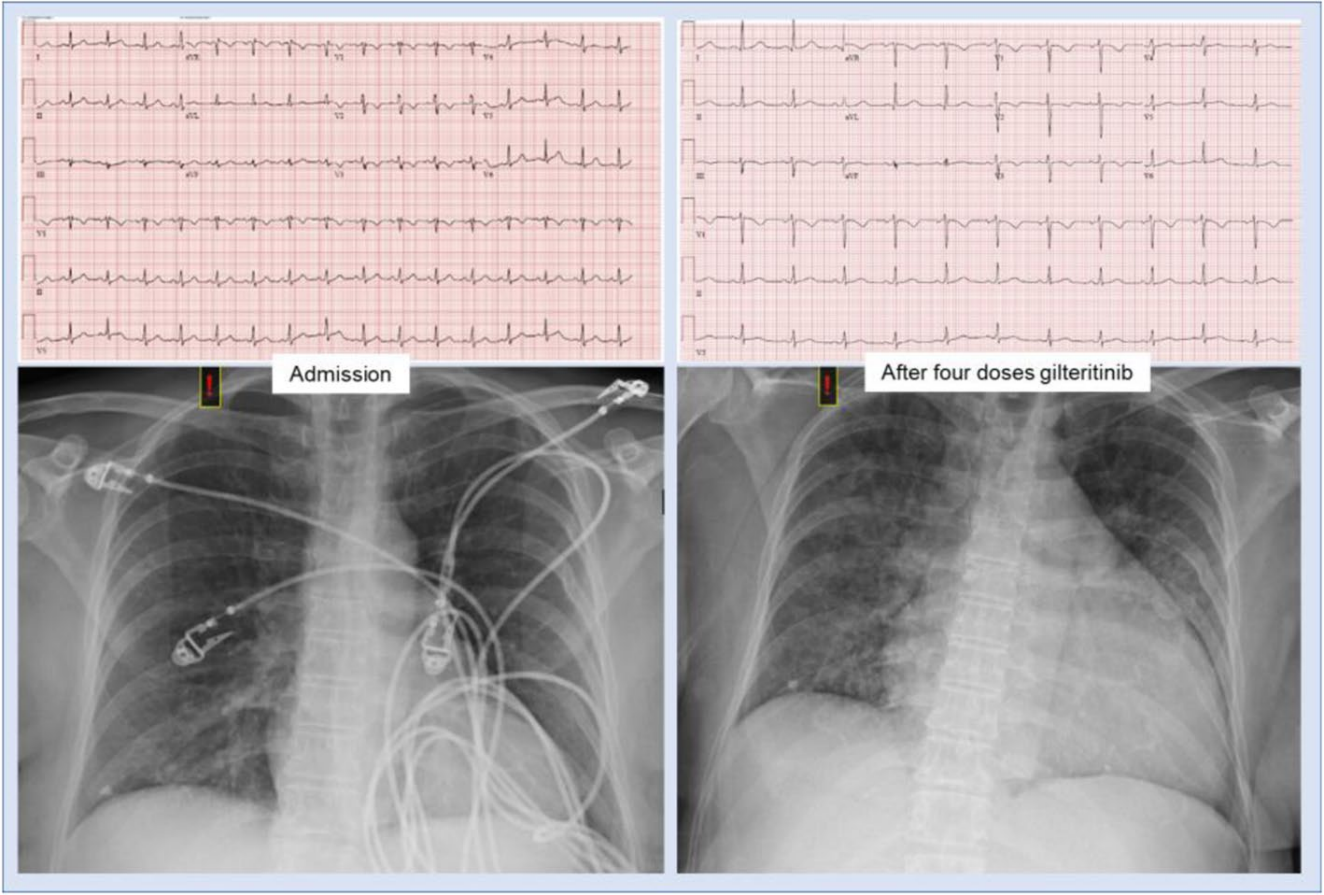
Admission electrocardiogram demonstrating sinus tachycardia (left upper) with negative T waves in leads V1-V3 and admission chest radiograph showing no acute cardiopulmonary disease (left lower). After four doses of gilteritinib and five days after admission, a repeat electrocardiogram is unremarkable (upper right). A repeat chest radiograph demonstrates interval cardiomegaly and diffuse pulmonary interstitial edema (lower right)

**Fig. 2:**
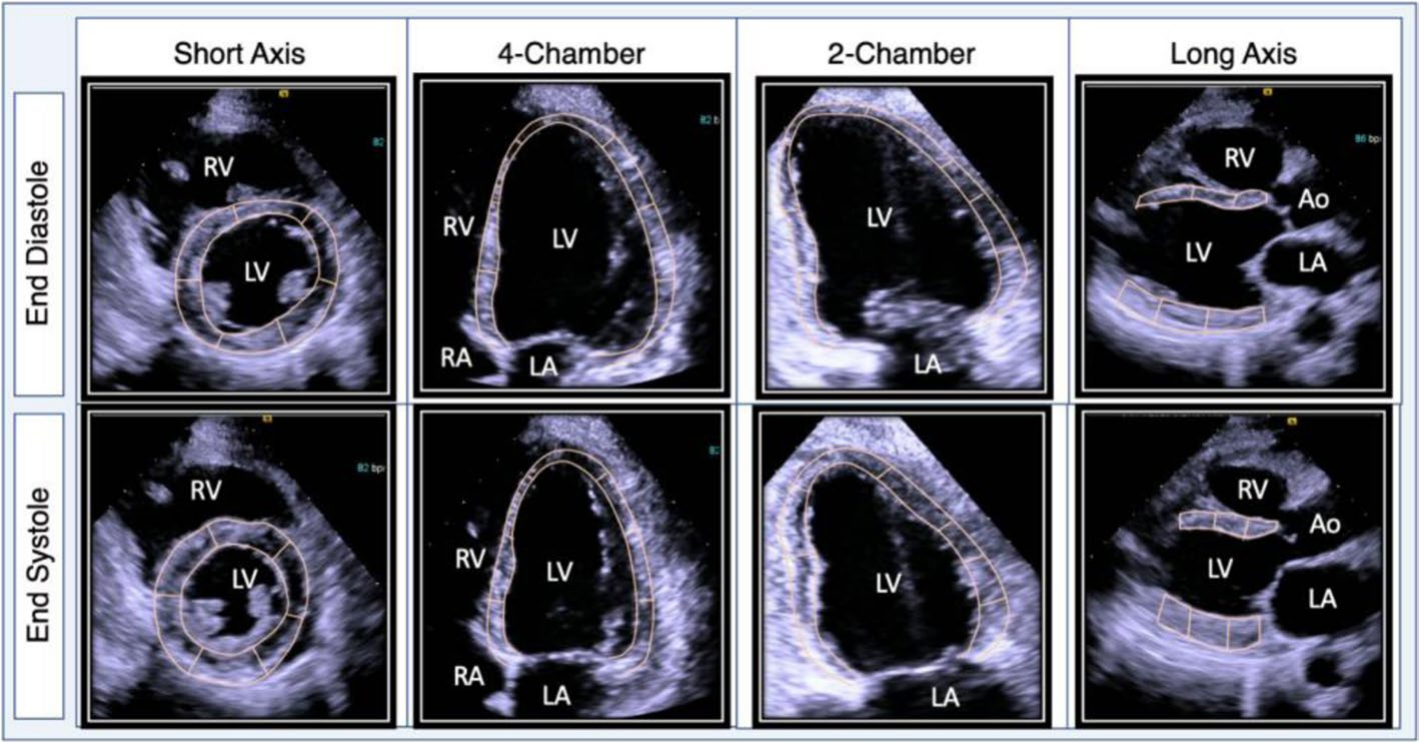
Echocardiogram after four doses of gilteritinib demonstrating acutely reduced systolic function and global hypokinesis. After four doses of gilteritinib targeted therapy, an echocardiogram showed moderately reduced left ventricular systolic function, an estimated ejection fraction of 36% with global hypokinesis and regional abnormalities of the septum

With additional laboratory studies, she was diagnosed with recurrent AML. Mutational analysis of her peripheral blood revealed new FLT3 internal tandem duplications (FLT3-ITD), a therapeutic target for AML. Before initiating targeted therapy, a baseline cardiac work-up was completed. Cardiac biomarkers demonstrated a mildly elevated brain natriuretic peptide 195 pg/mL (normal < 100 pg/mL) and undetectable troponin I < 0.11 ng/mL. An electrocardiogram (ECG) showed ST/T wave disturbances with negative T waves in leads V1-V3, perhaps suggesting prior anthracycline toxicity (Fig. 1). Transthoracic echocardiogram (TTE) showed a normal left ventricular ejection fraction (LVEF) of 67% and global longitudinal strain of − 24% (Fig. 2, Supplemental Movie 1 and 2).

The patient underwent cytoreductive therapy with hydroxyurea before transitioning to FLT3 targeted therapy with daily oral gilteritinib monotherapy. Following four doses of gilteritinib, she developed new shortness of breath, orthopnea, paroxysmal nocturnal dyspnea, bilateral lower extremity edema, and 23-pound weight gain. The examination was notable for new jugular venous distension, mild rhonchi, diminished breath sounds at the bilateral lung bases, and 2+ bilateral pitting edema of her lower extremities. Labs were notable for a decreased/improved white blood cell count to 0.87 K/uL with now 20% blasts, hemoglobin to 6.7 g/dL, and platelets to 49 K/uL. Brain natriuretic peptide was elevated to 1574 pg/mL and troponin I elevated to 0.36 ng/mL. Repeat chest radiography showed new cardiomegaly and worsening bilateral airspace disease compared to five days prior (Fig. 1). ECG was unchanged and did not reveal ischemic changes (Fig. 1). Repeat TTE ruled out pericardial effusion but demonstrated newly reduced LVEF to 35–40% from 67%, now with focal septal hypokinesis, new moderate to severe tricuspid regurgitation and elevation of right ventricular systolic pressure to 50 mmHg (Fig. 2, Supplemental Movie 3 and 4).

**Fig. 3:**
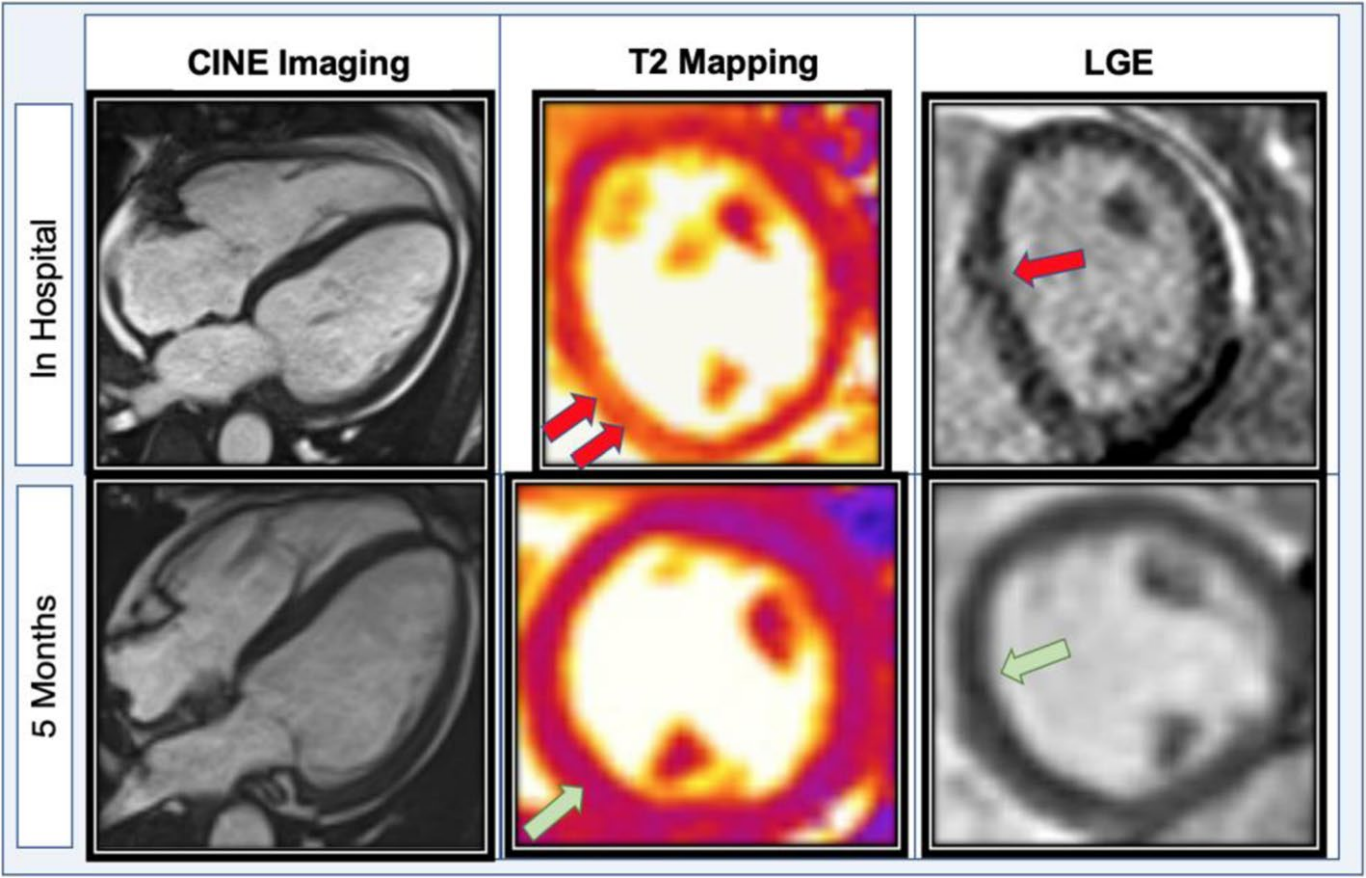
Cardiac magnetic resonance imaging (MRI) after six days of gilteritinib targeted therapy. After gilteritinib initiation, the patient developed significant clinical heart failure. Cardiac MRI revealed new left ventricular dilation and depressed systolic function with evidence of myocardial edema/ inflammation on T2 mapping (red arrows; max T2 of 70 milliseconds, normal < 53 ms), as well as patchy correlating non-ischemic patterned fibrosis with late gadolinium enhancement (LGE). Myocardial extracellular volume fraction was also elevated (not shown here). However, five months after therapeutic cessation, systolic function normalized with injury resolution (green arrows)

Gilteritinib was promptly discontinued after a total of four doses due to concern for acute cardiotoxicity. The patient progressed to acute hypoxic respiratory failure requiring eight L of supplemental oxygen and was treated with intravenous diuresis. Ischemic work-up with cardiac catheterization was deemed too high risk due to her active AML with pancytopenia. Cardiac magnetic resonance imaging (MRI) obtained two days after discontinuation of gilteritinib revealed borderline biventricular function with interval improvement in LVEF to 51%. Late-gadolinium enhancement imaging revealed patchy midwall fibrosis involving the septal and lateral walls and elevated T2 signal in the inferoseptum (up to 70 ms; normal < 53 ms) suggestive of myocardial edema or inflammation, findings which were consistent with inflammatory cardiomyopathy (Fig. 3, Supplemental Movie 5). Endomyocardial biopsy was not performed due to the associated procedural risks in this patient with relapsed AML.

Given the timecourse of events with new heart failure developing within days of treatment initiation, improved hematologic disease, and lack of alternative explanations, gilteritinib associated acute systolic heart failure and inflammatory cardiomyopathy was considered the most probable etiology due to the timeline of symptoms. Causality assessment of this suspected adverse drug reaction to gilteritinib is deemed probable/likely based on the Naranjo adverse drug reaction probability scale with a score of six [3]. Additionally, the timing of this clinical event occurred within a reasonable timeframe of drug administration. The clinical reaction abates after drug withdrawal or de-challenge [4]. Gilteritinib was withheld, and the patient eventually started guideline-directed medical therapy for non-ischemic cardiomyopathy with metoprolol succinate, lisinopril, and spironolactone once euvolemic. The patient declined rechallenge with gilteritinib. She continued to follow up with the AML transplant team and cardio-oncology team. She received two cycles of azacitidine and venetoclax before achieving morphologic and molecular remission of AML confirmed by bone marrow biopsy. Interval TTE five months later showed improved left ventricular function to 50–55% with mild global hypokinesis and reduced global longitudinal strain at − 17.6% (Supplemental Movie 6 and 7). Five months later, follow-up cardiac MRI showed recovered LV function with mild non-ischemic fibrosis and resolution of myocardial edema and inflammation on T2 mapping compared to prior (Fig. 3). Given this resolution of her myocarditis and AML remission, she received an allogeneic stem cell transplant from a matched unrelated donor.

## Discussion

To our knowledge, this case is the first reported event of acute systolic heart failure associated with the tyrosine kinase inhibitor gilteritinib in a patient without pre-existing cardiovascular disease. After four doses of gilteritinib, this patient abruptly developed symptoms of clinical heart failure with an acute reduction in LVEF to 35–40%. Further diagnostic evaluation with cardiac MRI showed mid-wall myocardial edema on T2-weighted imaging and late gadolinium enhancement in a non-coronary artery distribution, suggestive of inflammatory cardiomyopathy. Other differential diagnostic considerations include viral myocarditis, leukemic infiltration, or cardiac amyloidosis. However, the clinical time course and pattern of MRI findings were more suggestive that the drug may have precipitated her presentation. Gold standard definitive diagnosis with endomyocardial biopsy was deferred due to its associated procedural risks and low likelihood of altering management. Yet, given the increasing recognition of the potential induction of myocardial injury seen with several targeted or immunomodulatory therapies, cardiac MRI was promptly obtained [5]. Due to a lack of alternative diagnoses and a Naranjo adverse drug reaction probability scale of six, it is plausible to assign causation of this adverse drug reaction to gilteritinib [3]. This patient eventually recovered from symptomatic heart failure with stable LVEF after cessation of targeted therapy and the introduction of guideline-directed heart failure therapy. She was able to receive further cancer treatment successfully. In general, the onset, timeline, and likelihood of recovery from early cardiotoxicities associated with FLT3 inhibitors are unclear.

There is increasing evidence that FLT3 tyrosine kinase inhibitors are associated with cardiovascular toxicities, such as cardiomyopathies and QT prolongation in cancer clinical trials [6]. Currently, the FDA approved two FLT3 inhibitors, gilteritinib and midostaurin, for FLT3 mutated AML [2, 7, 8]. Gilteritinib gained approval after the phase III ADMIRAL trial, which demonstrated significantly higher overall survival (9.3 months vs. 5.6 months) and response rates (34% vs. 15.3%) in comparison with salvage chemotherapy in relapsed or refractory AML [2]. However, cardiac adverse events were observed among 138 enrollees within the first 30 days of therapy, including QT prolongation (7%), cardiac failure (4%), pericardial effusion (4%), pericarditis and myocarditis (2%) [7]. Similarly, midostaurin, a multi-kinase FLT3 inhibitor approved for combination with induction chemotherapy for newly diagnosed FLT3 mutated AML, was associated with QT prolongation (13%), leading to dose modification or treatment discontinuation [8]. Other adverse cardiac events in < 10% of patients included cardiac failure (6%) and myocardial infarction or ischemia (4%) in the initial trial, although the timeframe of these events is not specified [8]. With real-world clinical use, cardiotoxicities associated with emerging FLT3 inhibitors have become increasingly evident and may present unique clinical challenges regarding oncologic treatment decisions [6].

A mechanistic explanation of gilteritinib associated cardiotoxicity is currently limited as there is only one publication exploring the functional role of FLT3 signaling in cardiomyocytes. Pfister et al. showed that FLT3 and FLT3 ligand were both expressed in cardiomyocytes and proposed that activation of FLT3 signaling may serve as a cardioprotective anti-apoptotic system in the setting of oxidative stress and myocardial injury. In a mouse model of ischemia using LAD ligation, treatment with FLT3 ligand decreased infarction size, decreased apoptosis, and improved remodeling at the end of 1 week. They postulate that inhibition of FLT3 signaling in antileukemic targeted therapies may result in loss of this cardioprotective mechanism and lead to cardiomyocyte death, which may be a potential explanation for cardiotoxicity seen in FLT3 targeted cancer therapies [9]. Although the exact mechanism remains unclear, tyrosine kinase FLT3 inhibitors have been associated with significant cardiotoxicities, an important limitation in cancer therapy [6].

Currently, multiple clinical trials are exploring the role of gilteritinib in combination regimens [10]. However, in pivotal cancer clinical trials, the observed cardiovascular event rates used to guide most cardiotoxicity risk assessments during years of cumulative follow-up likely represent dramatic underestimates compared to clinical practice [6, 11]. Most cardiovascular events are not fully appreciated until real-world clinical application. Moving forward, baseline and ongoing cardiovascular surveillance are particularly important for monitoring early and late cardiotoxicity.

## Conclusion

This case demonstrates the potential for developing early cardiotoxicities with gilteritinib therapy, including non-ischemic cardiomyopathy and myocarditis, even in patients without pre-existing cardiovascular disease. The timeline and likelihood of reversibility of cardiac dysfunction from FLT3 inhibition remain unclear. Cardiac MRI can be an important modality to help differentiate or diagnose early cardiotoxicities linked with novel anticancer therapies. Clinicians are advised to obtain baseline and ongoing cardiovascular assessment in patients on targeted therapies for early recognition of potentially serious but rare cardiovascular complications of targeted therapy, particularly with acute change in clinical status.

## Supplementary Information

**Additional file 1.** Supplemental Movie 1: Baseline transthoracic echocardiogram, parasternal long axis view, demonstrating ejection fraction 67%

**Additional file 2.** Supplemental Movie 2: Baseline transthoracic echocardiogram, four chamber view – left ventricle focus, demonstrating ejection fraction 67%.

**Additional file 3.** Supplemental Movie 3: After 4 days of gilteritinib therapy, transthoracic echocardiogram, parasternal long-axis view, demonstrating ejection fraction 35–40%.

**Additional file 4.** Supplemental Movie 4: After 4 days of gilteritinib therapy, transthoracic echocardiogram, four-chamber view – left ventricle focus, demonstrating ejection fraction 35–40%.

**Additional file 5.** Supplemental Movie 5: Two days after gilteritinib cessation, cardiac MRI CINE, four-chamber view, demonstrating ejection fraction 51%.

**Additional file 6.** Supplemental Movie 6: Five months after gilterinib cessation, transthoracic echocardiogram, parasternal long-axis view, demonstrating ejection fraction 50–55%.

**Additional file 7.** Supplemental Movie 7: Five months after gilterinib cessation, transthoracic echocardiogram, four-chamber view – left ventricle focus, demonstrating ejection fraction 50–55%.

## Data Availability

All data generated or analyzed during this study are included in this published article and its supplementary information files.

## Abbreviations

TKI: Tyrosine kinase inhibitor
FLT3: FMS-like tyrosine kinase 3
AML: Acute myeloid leukemia
ECG: Electrocardiogram
TTE: Transthoracic echocardiogram
LVEF: Left ventricular ejection fraction
MRI: Magnetic resonance imaging
